# How can you survive a cardiac operation after cardiac operation? (analysis of pre- and postoperative data of redone cardiac operations)

**DOI:** 10.1186/1749-8090-8-S1-O130

**Published:** 2013-09-11

**Authors:** M Vaszily, B Matlakovics, R Paulinyi

**Affiliations:** 1 Hungarian Defense Forces; Health Center, Department of Cardio-Thoracic and Vascular Surgery, Budapest, Hungary

## Backgrund

The reoperations in cardiac surgery are technically more difficult, and the mortality and morbidity increased. It is huge importance of operative techniques, preoperative planning and the skills of the surgical team in those cases. We like to detect the different pre- and intra-operative reasons of the elevated morbidity and mortality, and the opportunities, how we could to reduce them.

## Methods

It was 79 REDO cardiac operations in our department during the last 2 years. The previous operations were: mitral valve (n=33), aortic valve (AVR) (n=15), CABG (n=22), AVR and CABG operations (n=4). We recorded the ejection fraction (EF), right ventricular pressure (RVP), intra-operative and postoperative complications, mortality, and postoperative bleeding.

## Results

The most common reason of major bleeding injuries (MBI) was the damage of right ventricle (25% of all MBI). In contrast to the literary data, in our cases the MBI were not increased the mortality, and the mean intensive care unit (ICU) time. The early ventricular fibrillation (13,6%), the MBI (36,4%) intra-arterial balloon pump application (13,6%) and mortality (18,2%) was higher after previous CABG operations. The incidence of non-fatal complications, MBI and the mortality were significant higher in those patients who had lower EF before, or/and higher RVP.

## Conclusion

The lower EF and higher RVP increases the mortality and elongated the mean ICU time. Due to this objects the REDO operation is indicated in the first time of the detection of necessity. The high RVP increase the possibility of MBI. In those cases the possible serious complications can be prevented by peripheral canulation, and the use of external defibrillator paddles. With proper preoperative planning and meticulous surgical techniques, the mortality and the serious postoperative complications can be reduced.

